# Pathophysiologic Basis and Diagnostic Approaches for Ischemia With Non-obstructive Coronary Arteries: A Literature Review

**DOI:** 10.3389/fcvm.2022.731059

**Published:** 2022-03-17

**Authors:** Bingqi Fu, Xuebiao Wei, Yingwen Lin, Jiyan Chen, Danqing Yu

**Affiliations:** ^1^Shantou University Medical College, Shantou, China; ^2^Division of Cardiology, Guangdong Cardiovascular Institute, Guangdong Provincial Key Laboratory of Coronary Heart Disease Prevention, Guangdong Provincial People's Hospital, Guangdong Academy of Medical Sciences, Guangzhou, China; ^3^Division of Geriatric Intensive Medicine, Guangdong Provincial Geriatrics Institute, Guangdong Provincial People Hospital, Guangdong Academy of Medical Sciences, Guangzhou, China

**Keywords:** ischemia with non-obstructive coronary arteries (INOCA), coronary microvascular dysfunction, coronary function test, diagnosis, pathophysiology, vasospasm

## Abstract

Ischemia with non-obstructive coronary arteries (INOCA) has gained increasing attention due to its high prevalence, atypical clinical presentations, difficult diagnostic procedures, and poor prognosis. There are two endotypes of INOCA—one is coronary microvascular dysfunction and the other is vasospastic angina. Diagnosis of INOCA lies in evaluating coronary flow reserve, microcirculatory resistance, and vasoreactivity, which is usually obtained *via* invasive coronary interventional techniques. Non-invasive diagnostic approaches such as echocardiography, single-photon emission computed tomography, cardiac positron emission tomography, and cardiac magnetic resonance imaging are also valuable for assessing coronary blood flow. Some new techniques (e.g., continuous thermodilution and angiography-derived quantitative flow reserve) have been investigated to assist the diagnosis of INOCA. In this review, we aimed to discuss the pathophysiologic basis and contemporary and novel diagnostic approaches for INOCA, to construct a better understanding of INOCA evaluation.

## Introduction

Ischemia with non-obstructive coronary arteries (INOCA) is defined as the stenotic reduction in diameter <50% detected by investigation of coronary arteries, either invasive coronary angiography (CAG) or coronary CT angiography (CCTA), in patients with symptoms of myocardial ischemia such as angina or dyspnea following physical activities (1–5). This particular population accounted for 70% of all the patients presenting with evidence of myocardial ischemia that eventually underwent CAG (6, 7). There are approximately 3–4 million American women and men who suffered from stable INOCA (8, 9). Epidemiologic analysis also demonstrated an evidently higher percentage of female patients that suffered from INOCA (10–12).

Strong pieces of evidence have shown that patients with INOCA have a higher risk of heart failure with preserved ejection fraction, major adverse cardiovascular events (MACEs), and mortality (4, 10, 13, 14). Compared to patients with no apparent coronary artery disease (no stenosis > 20%), patients with non-obstructive coronary arteries (≥1 stenosis ≥ 20% but no stenosis ≥ 70%) suffered a 2- to 4.5-fold higher risk of myocardial infarction, while the risk of myocardial infarction and mortality was similar to patients with single-vessel obstruction (stenosis ≥ 70% or left the main stenosis ≥ 50%) (13). The prevalence of coronary microvascular dysfunction (CMD), which is one of the major pathological mechanisms of INOCA, was as high as 75% in heart failure with preserved ejection fraction (15). The degree of severity in CMD was also associated with markers of heart failure severity, such as right ventricular dysfunction and N-terminal pro-B-type natriuretic peptide, and the rate of major adverse cardiovascular events (MACE) and heart failure hospitalization (15, 16). Particularly, female patients with INOCA face higher risks of unfavorable prognosis (8, 17–19). The first-year risk for MACE was 2.43 times higher in women with INOCA when compared to men (18). Apart from the above, many patients with INOCA encountered issues on quality of life, exercise capacity, and frequent hospital visits due to unsatisfying symptoms control (20).

The pathophysiological mechanisms of INOCA include CMD and/or epicardial vasospasm (21–23). Confirmatory diagnosis of INOCA requires an assessment of coronary microvasculature *via* invasive coronary routes (24). Fractional flow reserve (FFR), coronary flow reserve (CFR), index of microcirculatory resistance (IMR), hyperemic myocardial velocity resistance (HMR), and vasoreactivity in response to vasoactive agents are commonly used to assess the epicardial and microvascular conditions, and help to classify different endotypes of INOCA (1). However, such a technique is time-consuming and usually not widely available in some facilities, and therefore may not be able to meet the large demands of indicated candidates (25). Non-invasive methods that visualize myocardial perfusion, such as positron emission tomography (PET), can assess microvasculature more quickly (26). Also, there are some new techniques in invasive methods, such as continuous thermodilution and quantitative flow ratio (QFR), that are under research and are promising in complementing the diagnostic process of INOCA (27–29). In this review, we aimed to discuss the pathophysiologic basis and contemporary and novel diagnostic approaches for INOCA, to construct a better understanding of INOCA evaluation.

## Pathophysiologic Basis for Ischemia With Non-Obstructive Coronary Arteries

**Figure 1:**
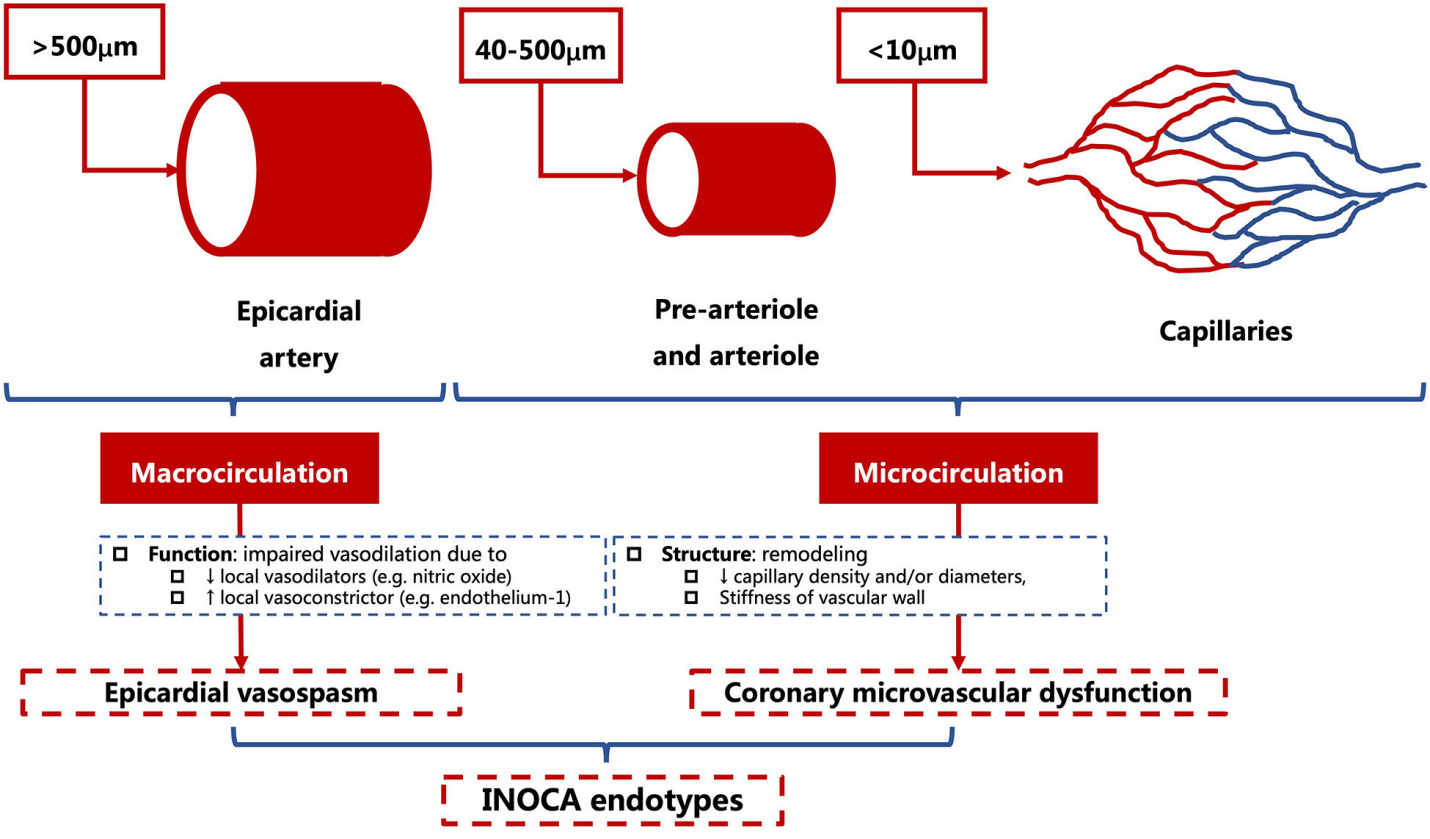
Anatomical structures and pathophysiological endotypes of INOCA. INOCA, ischemia with non-obstructive coronary arteries.

Coronary microcirculation refers to vessels smaller than 500 um in diameter, namely, the prearterioles (500–200 μm), arterioles (200–40 μm), and capillaries (<10 μm). The proximal segment to coronary microcirculation is the epicardial artery (500 μm to 5 mm), which is also known as the macrocirculation (Figure 1) (21, 30, 31). The pathophysiological mechanisms of INOCA include CMD and epicardial vasospasm that can overlap in some cases (21–23).

### Coronary Microvascular Dysfunction

The endothelial function is crucial in regulating the vascular tone of coronary microcirculation and maintaining cardiovascular homeostasis (32). During physical activity, increased oxygen consumption and local metabolites serve as triggers for the release of vasoactive substances, such as nitric oxide (NO) (33–36). NO has an anti-inflammatory function and plays a crucial role in protecting the integrity of the endothelium.

**Figure 2:**
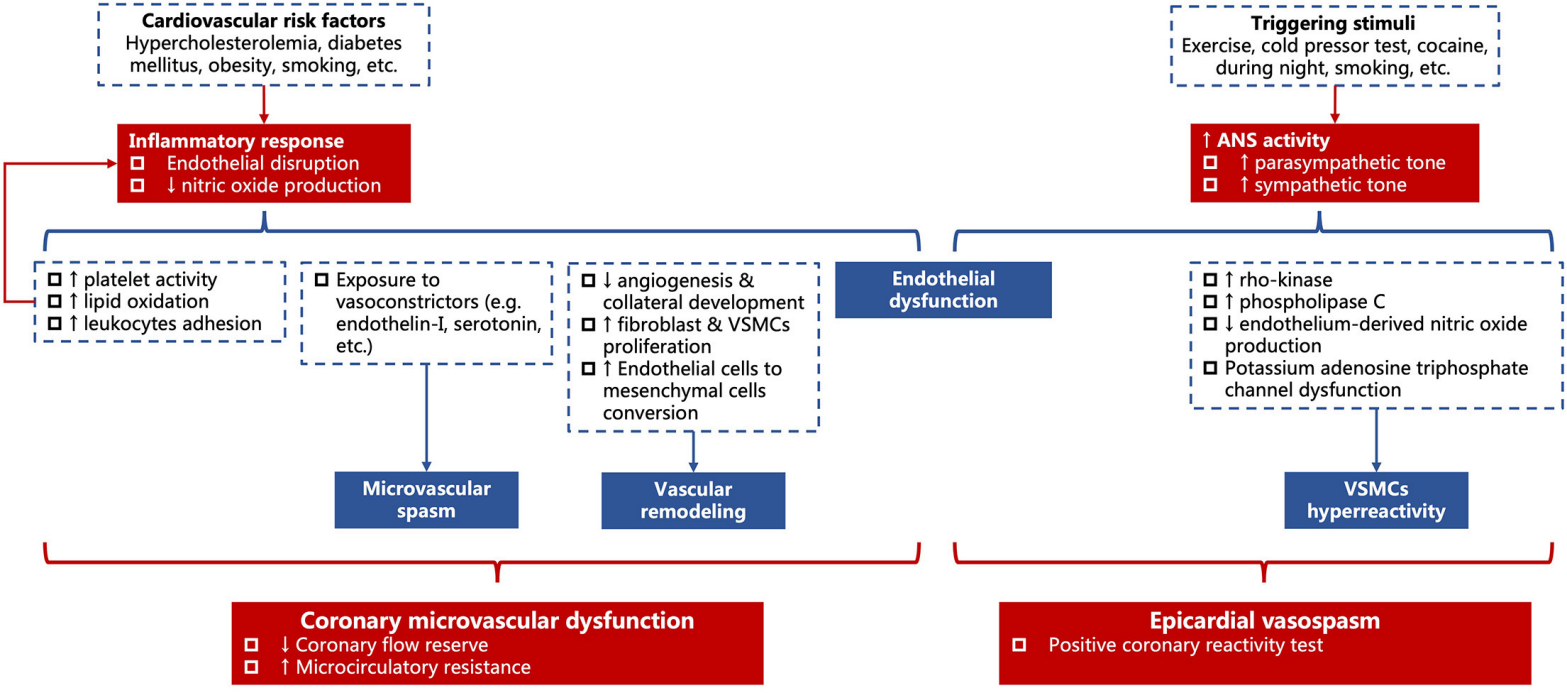
Diagram for the association between risk factors and hemodynamic changes in coronary microvascular dysfunction and epicardial vasospasm. VSMCs, vascular smooth muscle cells.

The structural (e.g., vascular remodeling, etc.) and functional abnormalities (e.g., endothelial dysfunction, microvascular spams, etc.) are proposed to be associated with the development of CMD. These mechanisms interact and are attributable for reduced coronary flow reserve and increased microcirculatory resistance (Figure 2) (37–39).

In response to common cardiovascular risk factors (e.g., hypercholesterolemia, diabetes mellitus, obesity, smoking, etc.), low-grade inflammation occurs and results in endothelium disruption and decreases the production of NO. The shortage of NO leads to increased platelet activity, lipid oxidation, and leukocytes adhesion, which, in turn, accelerates the inflammatory process (40, 41). It also impairs the process of angiogenesis and collateral development, meanwhile, promotes the proliferation of fibroblasts and vascular smooth muscle cells (VSMCs) and converts endothelial cells into mesenchymal cells (42, 43). The increased fibronectin and collagen deposition elongates the distance from capillary to adjacent cardiomyocytes and anticipates hypoxia on exertion (44–47). In addition, there is hypersensitivity toward the vasoconstrictor stimuli such as endothelin-I (48, 49) and serotonin (50), thus, a microvascular spasm might occur (Figure 2).

Chronic microvascular dysfunction is related to structural remodeling in patients with atherosclerosis, left ventricular hypertrophy cardiomyopathy, and arterial hypertension (21, 51, 52), where the arterial medial wall is thickened due to VSMCs proliferation and perivascular fibrosis, and the density of microvasculature is reduced (aka. microvascular rarefaction) due to diminished NO level (Figure 2) (51, 53, 54).

### Epicardial Vasospasm

Vasospasm can also occur in the epicardial artery (55). Common cardiovascular risk factors show no significant association with epicardial vasospasm, except for smoking (56, 57). Though inflammation and oxidative stress have been documented in vasospasm, they hardly constitute a major cause (56). Instead, the activity of the autonomous nervous system is important in the pathogenesis of epicardial vasospasm. Increased tone in the sympathetic nervous system [e.g., exercise (58), cold pressor test (59), and cocaine (60)] and/or parasympathetic nervous system [e.g., during the night (61)] can trigger epicardial vasospasm (Figure 2).

The two major mechanisms are endothelial dysfunction and VSMC hyperreactivity (57, 62).

Endothelium, as discussed before, can release NO to induce vasodilation. Normally, vasoactive stimuli (e.g., acetylcholine, serotonin, and histamine) mediate vasodilation *via* endothelium-dependent release of vasodilators, especially NO. When endothelium bears significant damage, these vasoactive stimuli might directly act on VSMCs and cause paradoxical vasoconstriction (Figure 2) (56, 63).

On the other hand, VSMC itself can become overreactive to vasoconstrictors, which is known as VSMC hyperreactivity. VSMC contraction or relaxation is controlled by rho-kinase myosin light chain phosphorylation or dephosphorylation (56, 64–66). However, there are multiple pathways involved in VSMC contraction, and theoretically, any disturbance on these pathways could lead to VSMC hyperreactivity. Increased activity of rho-kinase (65) and phospholipase C (67), disrupted endothelium-dependent release of NO (68), and potassium adenosine triphosphate channels dysfunction (69, 70) have been identified in epicardial vasospasm (Figure 2).

## Diagnostic Approaches for Ischemia With Non-Obstructive Coronary Arteries

Diagnostic approaches for INOCA are divided into non-invasive and invasive approaches, which have been studied extensively in recent years. The non-invasive approaches mainly include echocardiography, cardiac positron emission tomography (PET), single-photon emission CT (SPECT), CCTA, and cardiac MRI. The invasive approaches mainly include coronary function test (CFT) to measure CFR and/or microcirculatory resistance, coronary reactivity test to assess vasoreactivity, and some new-evolving techniques that facilitate these measurements (Table 1).

**Table 1:** Summary of diagnostic approaches of INOCA.

**Diagnostic approaches**	**Principle of measure**	**Parameters**	**Advantages**	**Disadvantages**
**Non-invasive methods**				
Stress transthoracic Doppler echocardiography	Flow velocity of mid-to-distal proportion of LAD	$\text{CF}(\text{V})\text{R=}\frac{\text{flow velocity at peak hyperemia}}{\text{flow velocity at rest }}$ (normal ≥2.5)	Non-invasive, no radiation, reproducible.	Lack of integration of the whole myocardium, high requirement for operators, inability to evaluate vasospasm.
Myocardial contrast echocardiography	Microbubbles that bounce echo signal in capillary beds, reflecting capillary blood volume	MBF = peak contrast intensity × myocardial flow velocity $\text{CFR=}\frac{\text{MBF at peak hyperemia}}{\text{MBF at rest }}$ (normal 4–5)	Non-invasive, no radiation, acceptable consistency with gold standard.	Variability, inability to evaluate vasospasm.
Cardiac positron emission tomography	Radiotracer labeled with isotopes allows myocardial perfusion imaging to perform	MBF obtained from radiotracer time-attenuation curve. $\text{MFR=}\frac{\text{MBF at peak hyperemia}}{\text{MBF at rest }}$ (normal >2.3)	Non-invasive, consistency with gold standard, accurate results, prognostic value.	Unavailability, expensiveness, radiation exposure, inability to evaluate vasospasm
Coronary computed tomography angiography	Iodine-contrast allows computed tomographic perfusion imaging to perform.	MBF obtained from contrast time-attenuation curve. (normal 75–164 ml/min/100 ml)	Non-invasive, supplementary	Rarely used in clinical practice, radiation exposure, inability to evaluate vasospasm
Cardiac magnetic resonance imaging	Gadolinium-based contrast allows first-pass perfusion imaging to perform	Myocardial perfusion obtained from time-attenuation curve. MFR index $\text{MFR index=}\frac{\text{myocardial perfusion at peak hyperemia}}{\text{myocardial perfusion at rest}}$ (normal > 1.5)	High-quality images, prognostic value, no radiation.	Unavailability, variability, expensiveness, time consumption.
**Invasive methods**				
Coronary function test	Wire-based technique (pressure, Doppler or bolus-thermodilution based methods) to assess coronary flow and microvascular resistance.	$\text{FFR=}\frac{\text{Pd at hyperemia}}{\text{Pa at hyperemia }}$ (normal >0.80) ${\text{CFR}}_{\text{Doppl}}=\frac{APV\;at\;hyperemia}{APV\;at\;rest}$ (normal ≥ 2.0) ${\text{CFR}}_{\text{thermo}}=\frac{Tmn\;at\;rest}{Tmn\;at\;hyperemia}$ (normal ≥ 2.0) IMR = Pd at hyperemia × Tmn at hyperemia (normal <25.0) $\text{HMR=}\frac{\text{Pd at hyperemia }}{\text{APV at hyperemia}}$ (normal <1.9)	Evaluation of epicardial and microvascular function, gold standard, prognostic value.	Invasive nature, operator variability.
Coronary reactivity test	Endothelium-dependent (acetylcholine, substance P, bradykinin) and endothelium-independent (adenosine, sodium nitroprusside) induction vasoreactivity	Epicardial spasm is diagnosed if (1) reproducible chest pain; (2) ischemic ECG shift; (3) more than 90% reduction of epicardial arterial diameter. Microvascular spasm is diagnosed if fitting (1), (2), and excluding (3)	Evaluation of vasospasm	Invasive nature, side effects of vasospasm.
Continuous thermodilution	Room temperature saline infusion at constant rate to achieve hyperemia at artery. Temperature change reflects coronary blood flow and resistance	$\text{AF=1}.\text{08 }\times \text{ Qi}\frac{\text{Ti}}{\text{T }}$ $\text{AR=}\frac{\text{Pd}}{\text{AF }}$ (normal range not available)	Operator-independent quantification, no side effects of vasodilators.	Normal range not available, interpersonal variability.
Angiography-derived quantitative flow reserve	Three-dimensional reconstruction of coronary angiography to evaluate epicardial artery	QFR is measured in mathematic models to compute FFR and A-IMR. (normal range not available)	Rapid and accurate results, non-invasive nature^a^, low cost.	Normal range not available

*INOCA, ischemia with non-obstructive coronary arteries; LAD, left anterior descending artery; CF(V)R, coronary flow (velocity) reserve; MBF, myocardial blood flow; MFR index, myocardial flow reserve index; FFR, fractional flow reserve; Pd, distal coronary pressure; Pa, aortic pressure; CFR_Doppl_, coronary flow reserve that measured by Doppler method; APV, average peak velocity; CFR_thermo_, coronary flow reserve that measured by thermodilution method; Tmn, mean transit time; IMR, index of microcirculatory resistance; HMR, hyperemic myocardial velocity resistance; ECG, electrocardiography; AF, absolute flow; Qi, infusion rate; Ti, temperature of saline; T, temperature of blood mixing with saline; AR, absolute resistance; QFR, quantitative flow ratio; A-IMR, angiography-derived index of microcirculatory resistance*.

a *QFR is in nature not invasive, but it requires invasive coronary angiography for 3D reconstruction*.

The 2019 European Society of Cardiology guideline for chronic coronary syndrome has clear recommendations for each of these diagnostic approaches. For patients with suspected CMD, that is, having persistent symptoms but coronary arteries that are either angiographically normal or have moderate stenoses with preserved FFR, the invasive approaches for measuring CFR and microcirculatory resistance should be considered, while the coronary reactivity test and non-invasive approaches for measuring CFR (i.e., echocardiography, cardiac PET, and cardiac MRI) may be considered. For patients with suspected vasospasm, that is, having a clinical picture of coronary spasm but normal findings or non-obstructive lesions on CAG, the coronary reactivity test should be considered (7).

## Non-Invasive Diagnostic Approaches

### Stress Transthoracic Doppler Echocardiography

Transthoracic color Doppler echocardiography is commonly performed at the mid-to-distal portion of the left anterior descending artery, and pulse wave Doppler is used to detecting flow velocity signal at rest and during hyperemia under stress. Stress can be induced by physical exercise (e.g., treadmill, exercise) or pharmacological agents (e.g., dobutamine, adenosine, dipyridamole) (71). Coronary flow velocity reserve [CF(V)R] is the ratio of coronary flow velocity during peak hyperemia and mean coronary flow velocity at rest (15, 72–76). A CFR <2.5 is defined as CMD (15, 77).

Though CFR is generally available and reproducible, its clinical use is limited by the lack of integration of the whole myocardium, in addition to the high requirement for operators (72, 73, 76, 78, 79).

### Myocardial Contrast Echocardiography (MCE)

Myocardial contrast echocardiography requires injection of ultrasonic contrast agent and microbubbles that have a similar size as red blood cells (<7 μm diameter). These microbubbles bounce echo signal, which is positively correlated with the capillary blood volume (80, 81). As 90% of coronary circulation is at capillaries at rest, a decayed signal intensity during continuous microbubble infusion would provide a hint of microvascular dysfunction (80, 82, 83). To obtain CFR, myocardial blood flow (MBF) needs to be measured by the product of peak contrast intensity (db) and myocardial flow velocity (db/s) in each myocardial segment first. Then, CFR is the ratio of MBF at peak hyperemia and MBF at rest. The normal interval of MCE-measured CFR was 4–5 (84).

Myocardial contrast echocardiography-measured CFR has been compared with the gold standard of CMD, which showed acceptable consistency. However, considerable variation of results among operators can disturb the reproducibility, which is due to the high variability of image quality and might be improved by increasing the numbers of operators (80–82). Therefore, quantitative and qualitative measurement of myocardial perfusion from MCE requires further validation (85).

### Cardiac PET

Cardiac PET is a technique based on the use of radiotracers labeled with isotope emission positrons. Common radiotracers are ^15^O-water, ^82^Rubidium, ^13^N-ammonia, and ^18^F-labeled agents (86). Their high myocardial extraction fraction allows the acquisition of myocardial perfusion images (MPI). MBF and myocardial flow reserve (MFR) are commonly obtained parameters (26). MBF is measured on dynamic PET. To measure MBF, “time-attenuation curves” that measure radioactive tracer activity transported by coronary arterial blood to myocardial tissue over time are required. The “time-attenuation curve” is fitted to a mathematic model, most commonly the one-tissue compartment model or simplified retention model. MBF is then estimated from the equation in the respective mathematic model (87–91). MFR is the ratio of MBF during pharmacological induced hyperemia to MBF at rest (86, 92) an MFR > 2.3 represents a favorable prognosis while an MFR <1.5 is suggestive of CMD and a high risk of future cardiac events (93, 94).

**Figure 3:**
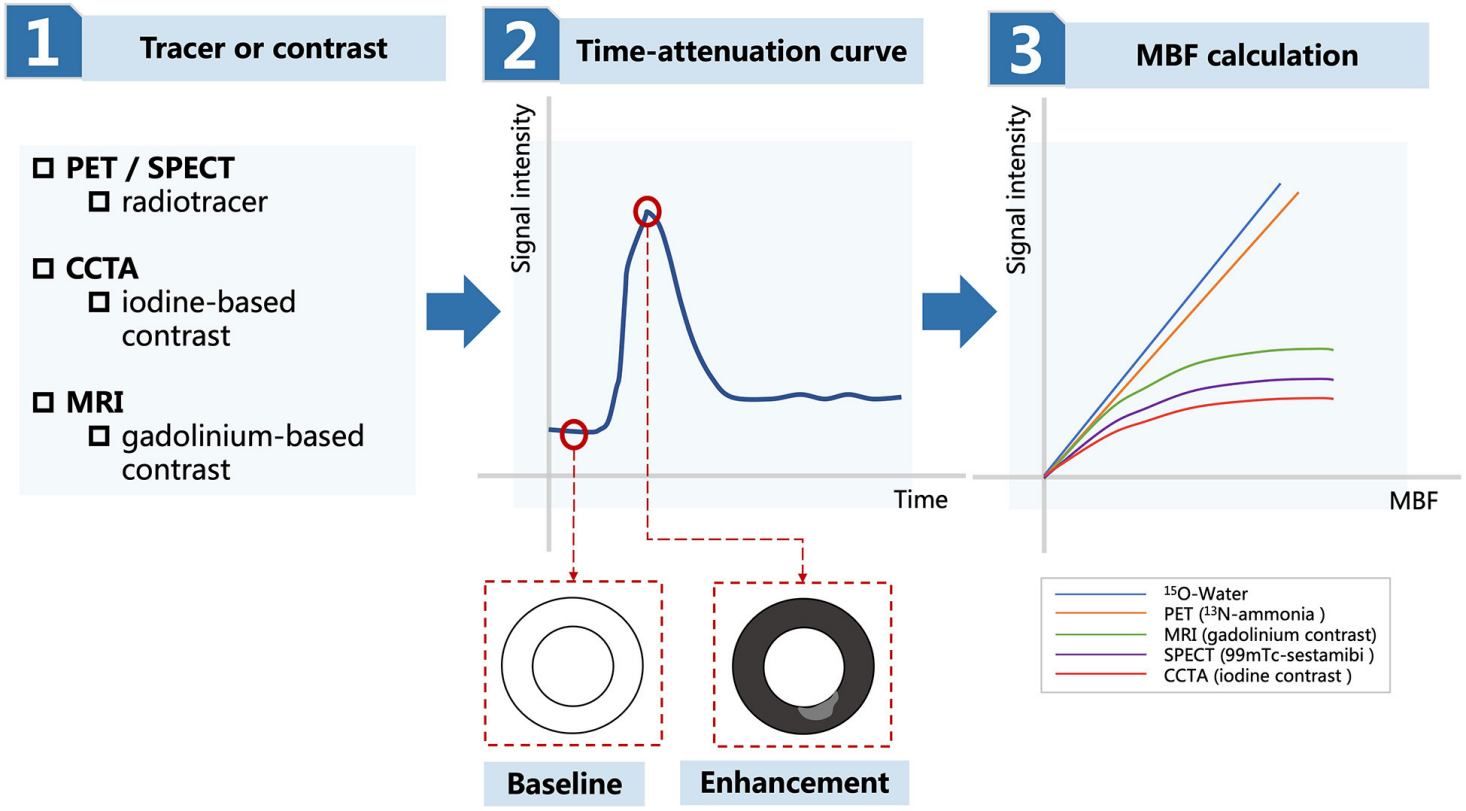
Diagram for the measurement of MBF in PET, SPECT, CCTA, and MRI. MBF, myocardial blood flow; PET, positron emission tomography; SPECT, single-photon emission computed tomography; CCTA, coronary computed tomography angiography; MRI, magnetic resonance imaging.

Cardiac PET is until now the most reliable method of non-invasive assessment of INOCA (74, 76, 91, 93, 95). The procedure is generally safe, time-saving, and accurate (96). The prognostic value has been verified for certain diseases, namely, hypertrophic cardiomyopathy (97), cardiac allograft vasculopathy (98), metabolic syndrome (99–101), heart failure with preserved ejection fraction (16), and female patients with INOCA (94), in which CMD plays an important role and impaired MFR is predictive of MACE (102, 103). However, PET is limited by its unavailability, cost-ineffectiveness, and radiation exposure even with a novel radiotracer (76, 93, 104). In addition, the cutoff value of MFR might vary among different PET tracers due to their unique kinetics (Figure 3) (105).

### Single-Photon Emission CT

Single-photon emission CT is a technique using different radioactive tracers from PET (i.e., 99mTc-sestamibi or 99mTc-tetrofosmin). MPI obtained from SPECT is used to estimate MBF and CFR (106). According to the “microsphere method” proposed by Sugihara et al. (107) it is assumed that radioactive tracers are taken up by myocardial tissue. MBF is the ratio of myocardial retention of tracers over the integral arterial concentration of tracers. CFR is the ratio of MBF during peak hyperemia to MBF at rest (108).

Compared to PET, SPECT has wider availability and MPI is more commonly performed (106). However, the estimation of SPECT-measured CFR was believed to be inaccurate, especially at the higher flow rate, and the optimal threshold was inconsistent across studies (Figure 3) (107, 109–111). Poor image quality is also a disadvantage due to tracer attenuation (106). To solve these problems, a cadmium-zinc-telluride semiconductor detector is a recently developed cardiac dedicated gamma camera system that can be used to reduce radiation exposure and improve time efficacy (112, 113). MPI obtained from dynamic SPECT reserves high quality of images and accuracy of data (108). MBF and CFR measurement from this cardiac dedicated SPECT are still under research, but the prognostic value of dynamic SPECT has been demonstrated (104, 114).

### Coronary CT Angiography (CCTA)

Coronary CT angiography is a commonly used screening test by using an iodine-based contrast agent to detect plaque and stenosis (106). However, CCTA is limited by the inability to identify ischemia, thus unable to provide diagnostic value for INOCA (7, 115). Dynamic computed tomographic perfusion image (CPI) is a recently developed technique that can be used to quantify MBF and other parameters. To calculate MBF, displacement of the myocardium is corrected and poor-quality images resulting from rhythm irregularities are excluded before data acquisition. Then, the ventricular myocardium is discretized into small volumetric elements, where each element creates their “time-attenuation curve,” respectively. The function of arterial input is generated by taking a sample volume in descending aorta. The arterial input function and “time-attenuation curve” are coupled in a hybrid deconvolution model. MBF is the ratio of the convoluted maximal slope of the “time-attenuation curve” of the myocardium and arterial input function (116). The normal range of MBF varies among studies: from 75 to 164 ml/min/100 ml (117). CFR is the ratio of MBF during hyperemia to MBF at rest (118). To calculate FFR, coronary blood flow simulation from CCTA images is required. Resting coronary blood flow is computed from myocardial territory-specific ventricular volume. Coronary resistance is computed from resting coronary flow according to the morphometry laws. Specifically, the microvascular resistance is inversely proportional to the size of vessels which carried a certain amount of blood (119, 120). Decrease in microvascular resistance in response to adenosine is modeled to simulate hyperemia. Mean aortic pressure is estimated from the mean brachial artery pressure. FFR is the ratio of computational mean coronary pressure distal to a lesion to the mean blood pressure in the aorta during simulated peak hyperemia (119–123).

The advance of CPI allows non-invasive quantification of FFR and CFR. Furthermore, the combination of CCTA and CPI can increase the diagnostic accuracy of INOCA and provide equivocal information with PET (124). Nevertheless, CPI is rarely used in clinical practice and its usefulness is yet to be tested in terms of INOCA (92, 106, 125).

### Cardiac MRI

Cardiac MRI is based on a gadolinium-based contrast agent and a first-pass perfusion image is commonly performed to assess myocardial ischemia. MBF can be obtained *via* visual assessment or semi-quantitative methods. As gadolinium first passes the myocardium, the signal intensity in the well-perfused myocardium is brighter than the ischemic area due to the shorter T1 relaxation time in the myocardium, which is visually evident. Without obstructive coronary arteries, diminished MBF might be related to CMD. MBF can also be quantified by the myocardial perfusion reserve (MPR) index (126–131). The myocardium is sliced into several segments. The signal intensity of the left ventricular blood pool and myocardium are measured. The myocardial upslope during contrast enhancement generates a “time-attenuation curve” which allows measurement of myocardial perfusion. The myocardial upslope is also normalized to adjacent ventricular blood pool upslope (132). MPR index is the ratio of myocardial perfusion during hyperemia to myocardial perfusion at rest. MPR index ≤ 1.5 is defined as pathological (126).

Myocardial perfusion reserve index has a significant correlation with CMD and shows good consistency with measurement from PET and invasive methods, and thus could be proposed as a non-invasive assessment tool for INOCA (131, 133–135). MPR index can be used in CMD for clinical outcome prediction, where one unit increase leads to 90% decrease in the risk of long-term MACE (136). Cardiac MRI has the advantages of high-resolution images and are free of radiation exposure, but can be limited by its cost ineffectiveness, time consumption, unavailability, and influence of baseline myocardial perfusion and tissue contrast concentration on the MPR index (Figure 3) (92, 106, 132).

### Summary for Myocardial Blood Flow Measurement in PET, SPECT, CCTA, and Cardiac MRI

The principle of measuring MBF in PET, SPECT, CCTA, and cardiac MRI is similar (Figure 3) (92, 106). The first step is to select a radiotracer in PET/SPECT or contrast agent in CCTA and cardiac MRI, which is then infused through vessels and taken up by the myocardium. The second step is to record myocardial signal intensity during enhancement and attenuation with time, which is illustrated as the “time-attenuation curve.” Well-perfused myocardium during enhancement is represented as the “black area” and ischemic myocardium during enhancement is represented as the “gray area.” The third step is to calculate MBF by fitting the “time-attenuation curve” to mathematic models. MBF estimation is related to signal extraction of the myocardium. There is a constant with 100% extraction for ^15^O-water and almost 100% extraction for ^13^N-ammonia. The extraction for agents commonly used in MRI, SPECT, and CCTA decreases as MBF increases, therefore underestimating MBF when MBF is high.

## Invasive Diagnostic Approaches in Inoca

### Coronary Function Test (CFT)

Coronary function test is a wire-based technique and is performed invasively when INOCA is considered after initial coronary angiography (7, 137). Multiple parameters are generated from this test, including but not limited to FFR, CFR, IMR, and HMR.

**Figure 4:**
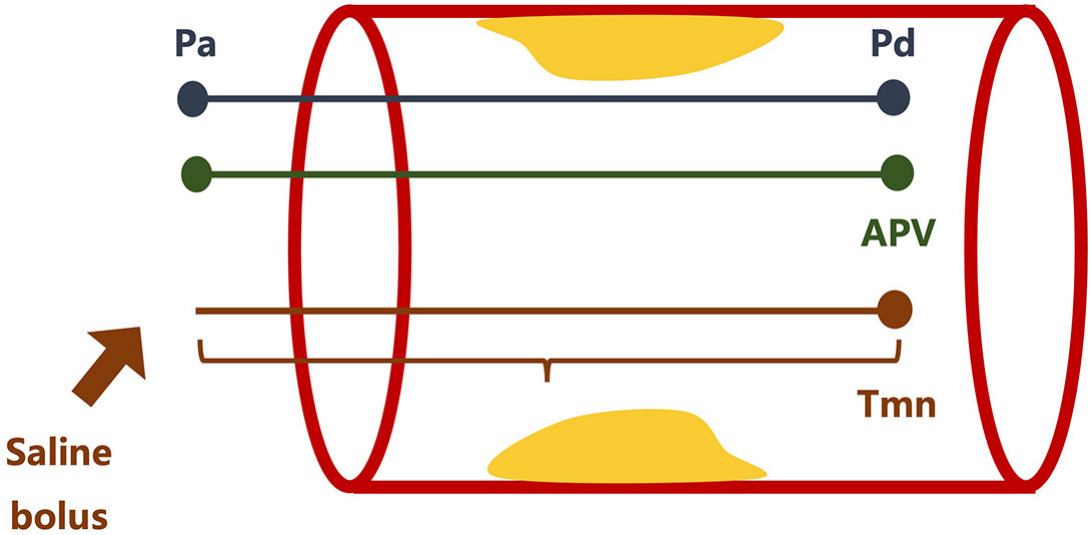
Illustration of coronary function test. Pd, distal coronary pressure; Pa, aortic pressure; APV, average peak velocity; Tmn, transit time.

With a guiding catheter placed at the ostium of the coronary artery, a pressure monitoring guide wire is calibrated and advanced distally to the stenotic lesion of the coronary artery. Hyperemia is induced by intravenous injection of a vasodilator such as adenosine. The distal coronary pressure (Pd) and aortic pressure (Pa) during maximal coronary flow are recorded. FFR is the ratio of Pd to Pa (Figure 4) (138, 139). Most of the recent studies defined 0.80 as the optimal threshold, and FFR ≤ 0.80 is indicated for PCI therapy (140–142). In the assessment of INOCA, FFR serves as the physiologic parameter of the epicardial artery and is particularly useful in distinguishing intermediate stenotic severity visualized on angiogram (143).

Coronary flow reserve (CFR) is the ratio of maximal to basal coronary flow. CFR can be measured *via* Doppler or thermodilution methods, and their equations to obtain CFR are slightly different. For the Doppler-based method, a Doppler guiding wire is used to measure phasic flow velocity and determine the average peak velocity (APV). CFR_Doppl_ is the ratio of hyperemic APV to resting APV (Figure 4) (144, 145). For the thermodilution method, a bolus of saline is injected manually into the guiding catheter and the mean transit time (*T*_mn_) is defined as the time needed for the bolus to travel from the guiding catheter to the sensor located in the distal part of the artery. CFR_thermo_ is the ratio of resting *T*_mn_ to hyperemic *T*_mn_ (Figure 4) (145–147). IMR is the product of Pd and *T*_mn_ during hyperemia *via* the thermodilution method (Figure 4) (148–150). HMR is the ratio of Pd to APV during hyperemia *via* the Doppler-based method (Figure 4) (151, 152). CFR is not as reproducible and specific as IMR owing to the influence of systemic hemodynamics (i.e., blood pressure, heart rate, etc.) and myocardial contractility. Therefore, IMR is usually necessary to assess microvascular resistance accurately (72, 153–155). CFR <2.0, IMR ≥ 25, and HMR ≥ 1.9 are defined as pathological and accounting for CMD (147, 154, 156–158).

**Figure 5:**
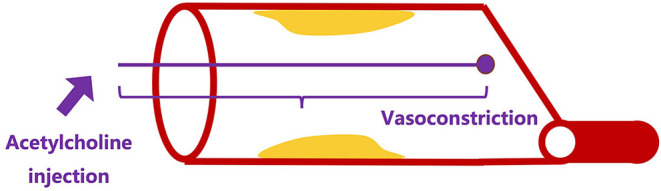
Illustration of coronary reactivity test.

A coronary reactivity test can be used to provide further information about vasospasm. Endothelial function was evaluated *via* endothelium-dependent (acetylcholine, substance P, and bradykinin) and endothelium-independent (adenosine and sodium nitroprusside) agents (1, 5, 159). Epicardial spasm is diagnosed if (1) reproducible chest pain, (2) detection of ischemic changes of electrocardiography, and (3) more than 90% reduction of epicardial arterial diameter. Microvascular spasm is diagnosed if (1) and (2) are present and excluding (3) (Figure 5) (160).

**Figure 6:**
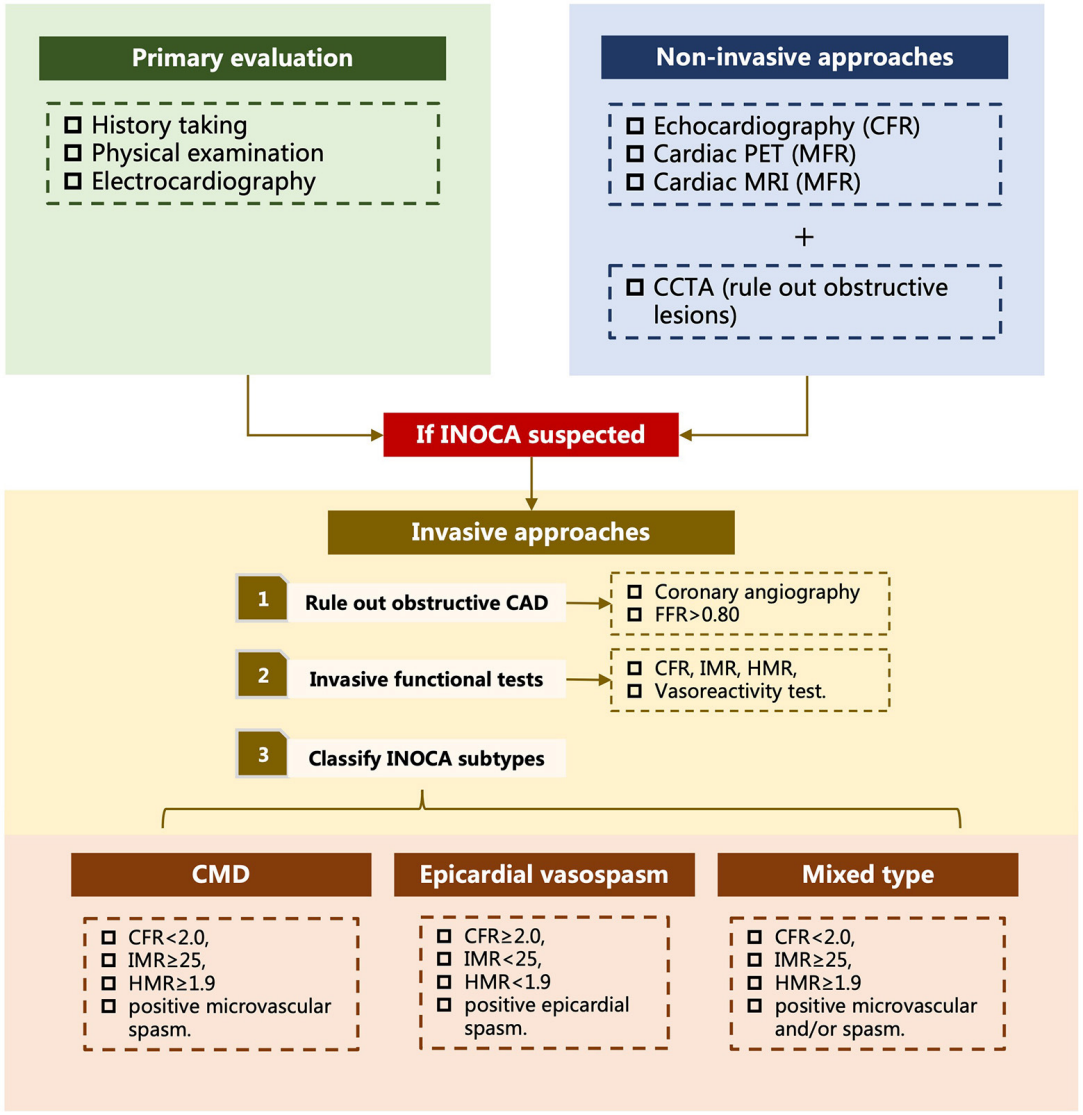
Diagram for the evaluation of INOCA. INOCA, ischemia with non-obstructive arteries; PET, positron emission tomography; MRI, magnetic resonance imaging; CCTA, coronary computed tomography angiography; CFR, coronary flow reserve; MFR, myocardial flow reserve; CAD, coronary artery disease; FFR, fractional flow reserve; IMR, index of microcirculatory resistance; HMR, hyperemic myocardial velocity resistance; CMD, coronary microvascular dysfunction. Mixed type refers to combination of coronary microvascular dysfunction and epicardial vasospasm.

The invasive evaluation of INOCA was proposed by the Coronary Vasomotion Disorders International Study Group. First of all, an initial assessment of the epicardial coronary artery is required to exclude an obstructive lesion, where FFR is strongly recommended in addition to the angiogram. Next, CFR, IMR, and HMR can be measured routinely to provide information regarding microvascular resistance. Last but not least, a coronary reactivity test is performed to reveal vasospasm. CMD is defined as FFR > 0.80, CFR <2.0, IMR ≥ 25, HMR ≥ 1.9, and positive microvascular spasm. Vasospastic angina is defined as FFR > 0.80, CFR ≥ 2.0, IMR <25, HMR <1.9, and epicardial spasm. Mixed type of CMD and vasospastic angina is defined as FFR > 0.80, CFR <2.0, IMR ≥ 25, HMR ≥ 1.9, and microvascular and/or epicardial spasm (Figure 6) (1, 24, 161, 162).

Besides confirmatory diagnosis and classification of INOCA, the role of CFT in predicting the prognosis of INOCA is widely acknowledged. Both CMD and epicardial vasospasm are relevant to MACE (163, 164). Patients with both CMD and epicardial vasospasm, which is defined as the mixed type of INOCA, have a particularly higher risk of unstable angina, myocardial infarction, and cardiac death (165, 166). CFT was also proven to be feasible in stratifying patients with INOCA for different treatment strategies (159, 167). Categorizing patients into different pathophysiological endotypes and treating them with specific protocol has improved symptom control of angina and quality of life to a significant extent (165, 167). Coronary blood flow changes in response to endothelium-dependent reactivity, i.e., acetylcholine infusion, which also has prognostic value. Coronary blood flow change of <50% is considered abnormal microvascular reactivity and is associated with long-term mortality (163). Non-invasive diagnostic approaches, when compared to CFT, showed extreme limitation in regards to detection of vasospasm, which is frequently encountered in INOCA (137). Therefore, CFT and coronary reactivity test are encouraged as soon as possible when INOCA is suspected, to prevent unnecessary diagnostic tests and promote early stratified treatment (137). The major limitation of CFT is its invasive nature and unavailability (25). However, the safety of invasive diagnostic procedures can be guaranteed by the experienced and dedicated interventional team and the rate of life-threatening complications such as coronary artery dissection, myocardial infarction, and ventricular arrhythmia are rare (168, 169). Overall, CFT is a safe and reliable diagnostic approach of INOCA.

### Continuous Thermodilution

Several disadvantages of Doppler and bolus thermodilution methods have been noticed, such as intraoperator variability and side effects resulting from vasodilators (115). Reliable results occurred only in 60–70% of the cases (170). Continuous thermodilution is a novel method that measures absolute flow (AF) and absolute resistance (AR) in a coronary artery, which is independent of operator quantification (28, 29). By infusing room temperature saline (*Ti*) at the rate of 8–20 ml/min (*Qi*), maximal hyperemia could be achieved in seconds (171, 172). Temperature of blood mixing with saline is denoted as *T*, which decreases as saline infused to the coronary artery. AF is the result of 1.08 × *Qi* × *Ti*/*T*.” AR is then calculated by dividing Pd by AF (28). Higher AR has been noticed to be associated with abnormal CFR and IMR, but neither AF nor AR is correlated with vasospasm (173). In addition, large interpersonal variability makes it difficult to establish the reference range of AF and AR. Further studies are required for assessing the validity of AF and AR (174).

### Angiography-Derived Quantitative Flow Ratio

Recently, a three-dimensional quantitative coronary angiography that enables rapid computation of FFR, which is denoted as QFR, has been developed. Coronary angiography is performed and two angiographic images that are at least 25 degrees apart from the artery of interest are selected. Three-dimensional reconstruction is automatically done and manual correction is allowed in case of suboptimal image quality. QFR can be calculated in three different flow models: (1) the fixed-flow QFR pullback: a fixed empiric hyperemic flow velocity of 0.35 m/s derived from Shengxian Tu et al. is used (27); (2) the contrast-flow QFR pullback: frame count analysis is performed on two diagnostic angiographic images without pharmacological induction. Modeled hyperemic flow velocity is derived by calculating two new QFR pullbacks (27); (3) the adenosine-flow QFR pullback: frame count analysis is performed on two diagnostic angiographic images with adenosine-induced hyperemia. Measured hyperemic flow velocity is derived by calculating two new QFR pullbacks (175). QFR shows good consistency with pressure wire-derived FFR and can be used to promote rapid computation of FFR (175–178). Furthermore, the formula of angiography-based IMR has been proposed. Without induction of hyperemia and use of pressure guidewire, angiography-based IMR also has a good correlation with IMR (179). QFR allows faster and more accurate evaluation of coronary microcirculation, therefore showing great potential in future practice. However, the reference range of FFR and IMR needs to be corrected in a larger sample size. CMD and acute coronary syndrome can influence the diagnostic performance of QFR. Therefore, indication for the use of QFR needs to be addressed before it becomes a routine tool in modern catheterization labs (179, 180).

## Discussion

Ischemia with non-obstructive coronary arteries is recognized as a clinical condition with strong evidence for unfavorable prognosis, including issues on quality of life, heart failure with preserved ejection fraction, MACE, and mortality. CMD and/or epicardial vasospasm are the two major pathophysiological mechanisms for INOCA. To establish a confirmatory diagnosis of INOCA, coronary blood flow, microvascular resistance, and vasoreactivity are required to be assessed, in which invasive coronary function test and coronary reactivity test are the gold standards. Several new techniques help complement the diagnosis of INOCA, such as continuous thermodilution and QFR. As for non-invasive methods, cardiac PET is a more reliable method compared to echocardiography, SPECT, CCTA, and cardiac MRI. Nevertheless, the non-invasive methods are unable to evaluate vasospasm, which is a frequently encountered endotype of INOCA. Another disadvantage is the variability of computed parameters due to poor image quality and low reproducibility.

A straightforward diagnostic diagram for INOCA evaluation has been proposed by Kunadian et al. (1). The evaluation of INOCA begins with history taking to address risk factors and events of ischemia, followed by physical examination and electrocardiography to exclude acute coronary syndrome at primary healthcare facilities. If patients are highly suspected to have INOCA, then they should be referred to cardiologists, where further diagnostic tests are performed. Non-invasive diagnostic approaches are the first-line tools. Techniques that can provide information about the coronary function (i.e., FFR, CFR, MBF, etc.) are preferred, such as echocardiography, cardiac PET, and cardiac MRI, based on local expertise and availability. CCTA may be performed to reveal any obstructive lesions on coronary arteries. According to the 2019 European Society of Cardiology guideline for the chronic coronary syndrome, invasive diagnostic approaches should be considered for patients with suspected CMD, that is, having persistent symptoms but coronary arteries that are either angiographically normal or have moderate stenoses with preserved FFR, or for patients with suspected vasospasm, that is, having a clinical picture of coronary spasm but normal findings or non-obstructive lesions on CAG (7). After ruling out obstructive CAD on CAG, invasive functional tests measuring CFR, IMR, HMR, and assessing vasoreactivity are performed to further classify INOCA into different endotypes, that is, CMD, epicardial spasm, and mixed type of CMD and epicardium (Figure 6).

In clinical practice, however, thorough diagnostic procedures on all suspected patients are unrealistic. Therefore, patients' follow-ups are essential for the assessment of the quality of life, identification of adverse events, prompt evaluation, and modification of treatment plans. In addition, though the confirmatory diagnosis has relied on invasive diagnostic approaches, non-invasive diagnostic approaches are promising as fast and accurate screening tests to meet the large demands of indicated candidates, if the limitations can be improved in future investigations. Last but not least, coronary microvascular dysfunction could be a regional presentation of systemic microvascular diseases. Diagnostic techniques that assess microvasculature in the kidney, retina, and cerebral white matter could also be an interesting research direction (181). The abbreviation and definition of this article are listed in Table 2.

**Table 2:** Abbreviation and definition.

**Abbreviation**	**Definition**
INOCA	Ischemia with non-obstructive coronary arteries
CMD	Coronary microvascular dysfunction
VSA	Vasospastic angina
CAG	Coronary angiography
MACE	Major adverse cardiovascular event
NO	Nitric oxide
VSMCs	Vascular smooth muscle cells
PET	Positron emission tomography
CCTA	Coronary computed tomography angiography
SPECT	Single-photon emission computed tomography
MRI	Magnetic resonance imaging
CFR	Coronary flow reserve
MCE	Myocardial contrast echocardiography
MBF	Myocardial blood flow
MPI	Myocardial perfusion image
MFR	Myocardial flow reserve
CPI	Computed tomographic perfusion image
MPR	Myocardial perfusion reserve
CFT	Coronary function test
FFR	Fractional flow reserve
IMR	Index of microcirculatory resistance
Pd^*^	Distal coronary pressure
Pa^*^	Aortic pressure
APV^*^	Average peak velocity
CFR_Doppl_	Coronary flow reserve that measured by Doppler method
CFR_thermo_	Coronary flow reserve that measured by thermodilution method
Tmn^*^	Mean transit time
HMR	Hyperemic myocardial velocity resistance
QFR	Quantitative flow ratio
AF	Absolute flow
AR	Absolute resistance

* *These abbreviations are used in equations and detailed definitions are included in the text*.

## Author Contributions

DY and JC conceived of the presented idea. BF wrote the manuscript. XW and YL revised the manuscript. All authors contributed to the article and approved the final version of the manuscript.

## Funding

This study was supported by grants from the Science and Technology Projects of Guangzhou (Grant No. 201903010097). The funders had no role in the study design, data collection and analysis, decision to publish, or preparation of the manuscript.

## Conflict of Interest

The authors declare that the research was conducted in the absence of any commercial or financial relationships that could be construed as a potential conflict of interest.

## Publisher's Note

All claims expressed in this article are solely those of the authors and do not necessarily represent those of their affiliated organizations, or those of the publisher, the editors and the reviewers. Any product that may be evaluated in this article, or claim that may be made by its manufacturer, is not guaranteed or endorsed by the publisher.
